# Refining Niche Metric Calculations: A Modified Weighting Approach to Colwell and Futuyma’s Method

**DOI:** 10.1007/s11538-026-01672-w

**Published:** 2026-06-12

**Authors:** Kürşad Özkan, Serkan Özdemir, Bart Muys, Salih Doğan

**Affiliations:** 1 Department of Forest Engineering, Faculty of Forestry, Isparta University of Applied Sciences, 32260 Isparta, Turkey; 2 Department of Forestry, Sütçüler Vocational School, Isparta University of Applied Sciences, 32950 Isparta, Turkey; 3 Division of Forest, Nature and Landscape, Department of Earth and Environmental Sciences, KU Leuven, 3000 Leuven, Belgium; 4 Department of Biology, Faculty of Science and Letters, Erzincan Binali Yıldırım University, 24002 Erzincan, Turkey

**Keywords:** Habitat niche, Ecological niche, Resource states, Niche breadth, Niche overlap, Noncircularity

## Abstract

The niche concept is central to ecology, evolution, and conservation biology. To assess resource dynamics in biological communities, the most widely used niche metrics are niche breadth and niche overlap. Colwell and Futuyma (1971) were the first to propose niche metrics that incorporated resource distinctness. In their framework, niche breadth and overlap are calculated using either a relative weighting factor $\left( {d_{j} } \right)$ or an absolute weighting factor $\left( {\delta_{j} } \right)$. Hanski (1978) later introduced an alternative weighting factor $\left( {\delta_{j}^{*} } \right)$, which accounts for both the quality and quantity of resource states in niche calculations. However, metrics based on $d_{j}$, $\delta_{j}$ and $\delta_{j}^{*}$ have inherent mathematical and conceptual limitations. To overcome these issues, the present study introduces modified forms of these factors ($ed_{j}$, $e\delta_{j}$ and $e\delta_{j}^{*}$) and evaluates their performance using eight hypothetical and one empirical (ecological) resource matrix. The findings indicate that niche metrics incorporating the $ed_{j}$ factor represent the most coherent and biologically meaningful option.

**Supplementary Information:**

The online version contains supplementary material available at 10.1007/s11538-026-01672-w.

## Introduction

The niche, one of the central concepts in ecology, evolution, and conservation biology, is widely used in studies that aim to explain the resource use, abundance, and distribution of organisms in space and time (Peterson et al. 2011; Carscadden et al. 2020). Three different definitions of the niche exist in the literature. The earliest definition was proposed by Joseph Grinnell (1917) and is referred to as the *Grinnellian niche*. This concept is based on the geographic distribution of species and the environmental factors that influence it, such as soil, topography, and climate. A second definition was introduced by Charles Elton and is known as the *Eltonian niche*. This concept focuses on species interactions, emphasizing the traits of organisms and their positions in the food web (Elton 1927). The Grinnellian and Eltonian niches cannot be investigated using the same tools, as they are often applied at different spatial and temporal scales (Soberón 2007; Hirzel and Le Lay 2008). The third definition was introduced by Hutchinson (1957). Hutchinson conceptualized the niche as an “n-dimensional hypervolume” defined by environmental conditions and resources required for a species to survive and maintain its population. Each dimension corresponds to a distinct variable, such as temperature, soil organic matter content, or nutrient availability. The Hutchinsonian niche is divided into two types: the *fundamental niche*, which describes the ideal environmental conditions under which a species can persist in the absence of competition, and the *realized niche*, which reflects the conditions under which a species persists when competition is present (Jiménez and Soberón 2022; Matthiopoulos 2022).

Quantitatively measuring the niche allows the identification of various ecological attributes such as species’ resource use, habitat preferences, and levels of competition. In this context, two fundamental metrics are most frequently used: niche breadth and niche overlap (Huang et al. 2025; Ni et al. 2024; Xiao et al. 2024; Pandey et al. 2023; Herrera-Lopera et al. 2022; Gu et al. 2017; Pierotti et al. 2017; Fu et al. 2015; Sá-Oliveira et al. 2013). Niche breadth reflects the extent to which a species exploits different types of resources (Krebs 2014). Species with broad niches are classified as generalists, whereas those with narrow niches are specialists (Pandey et al. 2023; Kovács and Carroll 2010). Ecologists have also referred to niche breadth as niche width*,* niche variation*,* tolerance*,* and ecological amplitude (Carscadden et al. 2020). Niche overlap, in contrast, refers to the degree to which two or more species use the same resources, and is important for understanding the potential intensity of interspecific competition (Krebs 2014). Beyond their classical role in community ecology, niche breadth and niche overlap metrics have become increasingly central to macroecological comparisons, climate change projections, functional trait analyses, and similarity-based diversity frameworks (Lancaster 2022; Zurell et al. 2024; Qiao et al. 2024). For instance, niche breadth has been employed to predict species’ range sizes and geographic distributions at global scales (Slatyer et al. 2013; Maitner et al. 2025), while niche overlap indices have been used to assess climate niche tracking and the consequences of range shifts in birds across continents (Zurell et al. 2024). Functional trait–based analyses have further extended the applicability of these metrics by linking resource use patterns to the morphological and physiological properties of organisms (Vivó-Pons et al. 2023; Huang et al. 2024).

The primary data source for calculating niche metrics is the resource matrix. A resource matrix is a dataset consisting of rows and columns that record the values of species’ resource use across different resource states (e.g., food types or habitat types). The accuracy of niche measurements depends directly on how resource states are defined and on the effectiveness of the sampling strategies employed. Colwell and Futuyma (1971) made a pioneering contribution to this field by developing a method for niche measurement based on resource matrices. In their approach, niche breadth and overlap are calculated using either a relative weighting factor $\left( {d_{j} } \right)$ or an absolute weighting factor $\left( {\delta_{j} } \right)$. However, two major criticisms have been raised regarding this method. The first criticism came from two related studies by Hanski and Koskela (1977) and Hanski (1978). Hanski and Koskela (1977) argued that the weighting factor $\delta_{j}$ proposed by Colwell and Futuyma (1971) does not account for distinctions between the qualitative and quantitative aspects of different resource states. Hanski (1978) further justified this criticism by showing that $\delta_{j}$ produces a high positive correlation with the abundance values of resource states. As a solution, he proposed a new weighting factor $\left( {\delta_{j}^{*} } \right)$ that considers the differential production of resource states. When actual productivity data are not available, Hanski suggested estimating this factor from total individual counts, following a similar assumption used by Sale (1974) in niche overlap calculations. The second, and more significant, criticism was raised by Loreau (1990). Using two hypothetical resource matrices, Loreau demonstrated that the assumption of neutrality among resource states in Colwell and Futuyma’s method can lead to inconsistent results, causing biases in niche estimates that increase with matrix size.

While the criticisms of Hanski (1978) and Loreau (1990) have long been acknowledged, the specific methodological consequences of these shortcomings for empirical applications deserve explicit attention. In practice, the existing weighting factors $d_{j}$, $\delta_{j}$, and $\delta_{j}^{*}$ suffer from three interrelated problems that compromise robust niche metric computation. First, certain matrix configurations—particularly those containing empty resource states or columns with zero total abundance—can cause weighting factors to become zero or mathematically undefined, rendering niche calculations impossible without ad hoc corrections. Second, when resource states are uniformly occupied across species, the resulting niche breadth and overlap values may be ecologically unintuitive, failing to distinguish between genuine generalist strategies and artefactual patterns produced by the weighting scheme itself. Third, the existing factors exhibit instability under matrix expansion: as the number of resource states increases through finer categorization, niche estimates may shift in magnitude and even in rank order among species, undermining the comparability of results across studies that differ in resource resolution. Taken together, these limitations are not merely theoretical curiosities but practical obstacles that can distort ecological inferences drawn from field data, especially in large-scale or multi-site comparisons where resource matrices inevitably vary in structure and dimensionality.

In parallel with these developments, several alternative frameworks for niche quantification have been proposed. De Cáceres et al. (2011) introduced a resemblance-based approach that incorporates the similarity between qualitative resources into niche metric calculations, thereby reducing the sensitivity of estimates to how resource categories are defined and subdivided. More recently, Hill number–based diversity measures have been increasingly advocated as a unifying statistical framework for biodiversity analysis, including applications to niche breadth and niche overlap (Alberdi and Gilbert 2019; Herrera-Lopera et al. 2022; Ricotta and Feoli 2024). These approaches express diversity in terms of effective numbers of equally abundant categories, providing intuitive and mathematically coherent indices. Furthermore, functional niche formulations based on continuous trait distributions have emerged as complementary tools for assessing niche differentiation among species (Mouillot et al. 2005; Vivó-Pons et al. 2023). Although the present study does not adopt these alternative frameworks directly, the proposed modifications to the Colwell and Futuyma (1971) weighting factors address a related concern: ensuring that niche metrics remain robust and ecologically interpretable regardless of resource matrix structure. By refining the information-theoretic approach from within, the present contribution complements these broader developments and offers a solution that preserves compatibility with the classical Colwell and Futuyma methodology while overcoming its known limitations.

This study was conducted with the aim of proposing improved weighting factors for the calculation of niche breadth and niche overlap, based on the pre-existing weighting factors $d_{j}$, $\delta_{j}$ and $\delta_{j}^{*}$, but taking into account the criticisms of Hanski (1978) and Loreau (1990) regarding the method of Colwell and Futuyma (1971), of testing these new weighting factors using hypothetical resource matrices, and of evaluating their applicability in niche metric calculations with real field data.

## Colwell and Futuyma Method

The niche metrics proposed by Colwell and Futuyma (1971) are modified forms of the most widely used niche breadth ($B_{i}$ and $B_{i}^{\prime}$) and niche overlap $C_{ih}$ and $S_{H}$) indices.

1 $$ B_{i} = \frac{1}{{\mathop \sum \nolimits_{j = 1}^{r} p_{ij}^{2} }} = \frac{{\left( {\mathop \sum \nolimits_{j = 1}^{r} N_{ij} } \right)^{2} }}{{\mathop \sum \nolimits_{j = 1}^{r} N_{ij}^{2} }} = \frac{{Y_{i}^{2} }}{{\mathop \sum \nolimits_{j = 1}^{r} N_{ij}^{2} }} $$

2 $$ B_{i}^{\prime} = - \mathop \sum \limits_{j = 1}^{r} p_{ij} \ln p_{ij} $$

3 $$ C_{ih} = 1 - \frac{1}{2}\left| {p_{ij} - p_{hj} } \right| $$

4 $$ S_{H} = \frac{{\mathop \sum \nolimits_{j = 1}^{r} \left( {p_{hj} + p_{ij} } \right)\ln \left( {p_{hj} + p_{ij} } \right) - \mathop \sum \nolimits_{j = 1}^{r} p_{hj} \ln p_{hj} - \mathop \sum \nolimits_{j = 1}^{r} p_{ij} \ln p_{ij} }}{2\ln 2} $$

Here, $B_{i}$, $B_{i}^{\prime}$, $C_{ih}$ and $S_{H}$ represent, respectively, Levins’ index (the reciprocal of Simpson’s dominance index), Shannon entropy $\left( {B_{i}^{\prime} } \right)$ (Levins 1968; Shannon 1948; Simpson 1949), the Czekanowski index (Mimoto and Zitikis 2009; Colwell and Futuyma 1971), and Horn’s index (Horn 1966; Medina-Nava et al. 2011; Pierotti et al. 2017). The notations used in Eqs. (1)–(4) and the niche metrics of Colwell and Futuyma (1971) are given in Table 1.

**Table 1:** Notation for the resource matrix

	Resource states					
Species	$N_{11}$	…	$N_{1j}$	…	$N_{1r}$	${\boldsymbol{Y}}_{1}$
	⋮		⋮		⋮	
	$N_{i1}$	…	$N_{ij}$	…	$N_{ir}$	${\boldsymbol{Y}}_{{\boldsymbol{i}}}$
	⋮		⋮		⋮	
	$N_{s1}$	…	$N_{sj}$	…	$N_{sr}$	${\boldsymbol{Y}}_{{\boldsymbol{s}}}$
	${\boldsymbol{X}}_{1}$	**…**	${\boldsymbol{X}}_{{\boldsymbol{j}}}$	**…**	${\boldsymbol{X}}_{{\boldsymbol{r}}}$	${\boldsymbol{Z}}$

The matrix is composed of *r* resource states and *s* species. $$N_{ij}$$ denotes the abundance of species *i* in resource state *j*; $$X_{j}$$ is the total abundance of resource state *j* (column total); $$Y_{i}$$ is the total abundance of species *i* (row total); and $$Z $$ is the grand total abundance in the data matrix

Table 1 provides a representative illustration of a resource matrix. This matrix is composed of $r$ resource states and $s$ species. Resource matrix may represent food types or habitat types (De Cáceres et al. 2011).

From the resource matrix, $\pi_{ij}$ is derived according to Eq. (5). $\pi_{ij}$ denotes the estimated probability that a randomly selected individual from the pooled community belongs to species $i$ and is associated with resource state $j$. The conditional probabilities are defined by Eqs. (6)–(9):

5 $$ \pi_{ij} = \frac{{N_{ij} }}{Z} $$

6 $$ p_{ij} = \frac{{N_{ij} }}{{Y_{i} }} $$

7 $$ P_{j} = \frac{{X_{j} }}{Z} $$

8 $$ q_{ij} = \frac{{N_{ij} }}{{X_{j} }} $$

9 $$ Q_{i} = \frac{{Y_{i} }}{Z} $$

In this framework, $p_{ij}$ represents the probability that an individual utilizes resource state $j$, conditional on its membership in species $i$, whereas $q_{ij}$ represents the probability that an individual belongs to species $i$, conditional on its association with resource state $j$. $Q_{i}$ corresponds to the marginal probability of an individual belonging to species $i$, irrespective of resource state, while $P_{j}$ denotes the marginal probability of an individual being associated with resource state $j$, independent of species identity. Accordingly, the probability distributions satisfy the normalization condition: $\mathop \sum \nolimits_{i = 1}^{s} \mathop \sum \nolimits_{j = 1}^{r} \pi_{ij} = \mathop \sum \nolimits_{j = 1}^{r} p_{ij} = \mathop \sum \nolimits_{i = 1}^{s} q_{ij} = \mathop \sum \nolimits_{j}^{r} P_{j} = \mathop \sum \nolimits_{i}^{s} Q_{i} = 1$.

The method proposed by Colwell and Futuyma (1971) for calculating niche metrics is grounded in information theory. In this framework, niche-related probabilities are quantified using entropy functions. Specifically, the marginal entropies of resources and species $H\left( X \right)$, $H\left( Y \right)$, and their joint entropy $H\left( {XY} \right)$, are defined by Eqs. (10)–(12). Conditional entropy, $H_{Y} \left( X \right)$, is then obtained as in Eq. (13).

10 $$ H\left( X \right) = - \mathop \sum \limits_{j = 1}^{r} P_{j} \ln P_{j} $$

11 $$ H\left( Y \right) = - \mathop \sum \limits_{i = 1}^{s} Q_{i} \ln Q_{i} $$

12 $$ H\left( {XY} \right) = - \mathop \sum \limits_{i = 1}^{s} \mathop \sum \limits_{j = 1}^{r} \pi_{ij} \ln \pi_{ij} $$

13 $$ H_{Y} \left( X \right) = H\left( {XY} \right) - H\left( Y \right) $$

Alternative formulations of $H_{Y} \left( X \right)$ include $- \mathop \sum \nolimits_{i = 1}^{s} \mathop \sum \nolimits_{j = 1}^{r} \pi_{ij} \ln p_{ij}$ or $- \mathop \sum \nolimits_{i = 1}^{s} Q_{i} H_{i} \left( X \right)$, where $H_{i} \left( X \right) = - \mathop \sum \nolimits_{j = 1}^{r} p_{ij} \ln p_{ij}$.

Joint entropy $\left( {m\left( X \right)} \right)$ provides a basis for calculating standardized resource heterogeneity $\left( {M\left( X \right)} \right)$ (Eqs. 14, 15). The contribution of each resource state, $M_{j} \left( X \right)$, is defined in Eq. (16), while relative weights $d_{j}$ are obtained from Eq. (17), ensuring that $\mathop \sum \nolimits_{j = 1}^{r} d_{j} = 1$.

14 $$ m\left( X \right) = m\left( Y \right) = H\left( X \right) - H_{Y} \left( X \right) = H\left( Y \right) - H_{X} \left( Y \right) $$

15 $$ M\left( X \right) = \frac{m\left( X \right)}{{H\left( X \right)}} = \frac{{H\left( X \right) - H_{Y} \left( X \right)}}{H\left( X \right)} = 1 - \frac{{H_{Y} \left( X \right)}}{H\left( X \right)} $$

16 $$ M_{j} \left( X \right) = \delta_{j} = \frac{{\mathop \sum \nolimits_{i = 1}^{s} \mathop \sum \nolimits_{j = 1}^{r} \pi_{ij} \ln p_{ij} - P_{j} \ln P_{j} }}{H\left( X \right)} $$

17 $$ d_{j} = \frac{{M_{j} \left( X \right)}}{{\mathop \sum \nolimits_{j = 1}^{r} M_{j} \left( X \right)}} = \frac{{\delta_{j} }}{{\mathop \sum \nolimits_{j = 1}^{r} M_{j} \left( X \right)}} $$

Weighted values of resource states $\left( {d_{j} } \right)$ allow the computation of adjusted abundances $Y_{i}^{*}$ and corresponding probabilities $p_{ij}^{*}$ (Eqs. 18, 19).

18 $$ Y_{i}^{*} = \mathop \sum \limits_{j = 1}^{r} d_{j} kN_{ij} $$

19 $$ p_{ij}^{*} = \frac{{N_{ij} }}{{\mathop \sum \nolimits_{j = 1}^{r} d_{j} kN_{ij} }} = \frac{{N_{ij} }}{{Y_{i}^{*} }} $$

Here $k$ denotes the constant parameter selected for the purpose of matrix expansion.

The niche breadth $\left( {B_{i} } \right)$ and standardized niche breadth $\left( {\beta_{i} } \right)$ based on Levin’s index (Eq. 1) are calculated using Eqs. (20) and (21), whereas the niche breadth $\left( {B_{i}^{\prime} } \right)$ and standardized niche breadth $\left( {\beta_{i}^{\prime} } \right)$ based on Shannon entropy (Eq. 2) are determined using Eqs. (22) and (23).

20 $$ B_{i} = \frac{1}{{\mathop \sum \nolimits_{j = 1}^{r} d_{j} kp_{ij}^{*2} }} $$

21 $$ \beta_{i} = \left[ {\frac{1}{{\mathop \sum \nolimits_{j = 1}^{r} d_{j} kp_{ij}^{*2} }} - 1} \right]\left[ {\frac{1}{k - 1}} \right] $$

22 $$ B_{i}^{\prime} = - \mathop \sum \limits_{j = 1}^{r} d_{j} k\left( {p_{ij}^{*} \ln p_{ij}^{*} } \right) $$

23 $$ \beta_{i}^{\prime} = - \frac{k}{\ln k}\mathop \sum \limits_{j = 1}^{r} d_{j} \left( {p_{ij}^{*} \ln p_{ij}^{*} } \right) $$

Species with the lowest values of $B_{i}^{\prime}$ or $\beta_{i}^{\prime}$ are considered specialists, whereas those with the highest values are generalists. When all resource states are used equally, $B_{i}^{\prime} = { }\beta_{i}^{\prime} = 1$.

Colwell and Futuyma (1971) proposed two modified formulas for calculating niche overlap. The first, $\gamma_{ih}$, is a modification of the $C_{ih}$ formula presented in Eq. (3):

24 $$ \gamma_{ih} = 1 - \frac{1}{2}\mathop \sum \limits_{j = 1}^{r} d_{j} k\left| {p_{ij}^{*} - p_{hj}^{*} } \right| = \mathop \sum \limits_{j = 1}^{r} d_{j} - \frac{1}{2}\mathop \sum \limits_{j = 1}^{r} d_{j} k\left| {p_{ij}^{*} - p_{hj}^{*} } \right| $$

The second niche overlap measure, $\gamma_{ih}^{\prime}$, is based on Horn’s index $\left( {S_{H} } \right)$ given in Eq. (4). An alternative formulation of $S_{H}$ is:

25 $$ C_{ih}^{\prime} = S_{H} = - \frac{1}{2\ln 2}\mathop \sum \limits_{j = 1}^{r} \left[ {I\left( {p_{ij} } \right) + I\left( {p_{hj} } \right) - I\left( {t_{j} } \right)} \right] $$

where $I\left( x \right) = x\ln x$, $t_{j} = p_{ij} + p_{hj}$, $p_{ij} = N_{ij} /Y_{i}$, and $p_{hj} = N_{hj} /Y_{h}$. The value of $C_{ih}^{\prime}$ ranges from 0 to 1: it equals 0 if species $i$ and $h$ occupy entirely different resource states, and equals 1 if they share identical proportional distributions across the same resource states.

The formula $\gamma_{ih}^{\prime}$ is a modified version of $C_{ih}^{\prime}$, where $p_{ij}^{*}$ is determined using Eq. (19):

26 $$ \gamma_{ih}^{\prime} = - \frac{1}{2\ln 2}\mathop \sum \limits_{j = 1}^{r} d_{j} k\left[ {I\left( {p_{ij}^{*} } \right) + I\left( {p_{hj}^{*} } \right) - I\left( {t_{j}^{*} } \right)} \right] $$

Here, $$t_{j}^{*} = p_{ij}^{*} + p_{hj}^{*}$$, $$p_{ij}^{*} = N_{ij} /Y_{i}^{*}$$ and $$p_{hj}^{*} = N_{hj} /Y_{h}^{*}$$.

Although the constant $$k$$ does not influence the estimation of niche overlap, it plays a critical role in determining niche breadth values. Therefore, the same $$k$$ value should be applied consistently within a given analysis to ensure comparability across species. Colwell and Futuyma (1971) recommended a value of 10,000 as an appropriate standard for entomological field datasets. Nonetheless, $$k $$ must be substantially greater than $$r$$ to prevent the occurrence of positive logarithmic values in Eq. (23).

## Modified Versions of the Colwell and Futuyma Method

Three studies have drawn attention in the literature for their modified applications of the Colwell and Futuyma (1971) method: Clarke (1977), Hanski (1978), and Alatalo (1981).

Clarke (1977) followed the Colwell and Futuyma approach but modified it by excluding the $k$ parameter instead of using the $\beta_{i}^{\prime}$ formula, calculating species’ niche breadths across different habitats, referred to as “habitat niche breadth.” In this approach, the adjusted probabilities $p_{ij}^{*}$ and niche breadth $W_{i}^{\prime}$ are defined as:

27 $$ p_{ij}^{*} = \frac{{N_{ij} }}{{\mathop \sum \nolimits_{j = 1}^{r} d_{j} N_{ij} }},\;\mathop \sum \limits_{j = 1}^{r} d_{j} p_{ij}^{*} = 1 $$

28 $$ W_{i}^{\prime} = \exp \left( { - \mathop \sum \limits_{j = 1}^{r} d_{j} p_{ij}^{*} \ln p_{ij}^{*} } \right) $$

Clarke also calculated niche overlap based on $p_{ij}^{*}$, using a formula equivalent to Colwell and Futuyma’s $\gamma_{ih}$:

29 $$ O_{ih}^{\prime} = 1 - \frac{1}{2}\mathop \sum \limits_{j = 1}^{r} d_{j} \left| {p_{ij}^{*} - p_{hj}^{*} } \right| $$

Hanski (1978) highlighted a strong positive correlation between the abundance of resource states $\left( {X_{j} } \right)$ and the absolute weighting factor $\left( {\delta_{j} } \right)$ of Colwell and Futuyma (1971), noting that this correlation could introduce biases in niche metric calculations. To reduce these biases, he proposed a new factor $\left( {\delta_{j}^{*} } \right)$ based on $\delta_{j}$ and the abundance of resource states:

30 $$ \delta_{j}^{*} = \left( {\delta_{j} /X_{j} } \right)/\mathop \sum \limits_{i = 1}^{r} \left( {\delta_{j} /X_{j} } \right) $$

Alatalo (1981) used the same $p_{ij}^{*}$ formula as Colwell and Futuyma, but introduced a modified niche breadth formula $\left( {B_{A} } \right)$ incorporating $k$ and $d_{j}$, where variations in $k$ do not affect the results:

31 $$ p_{ij}^{*} = \frac{{N_{ij} /d_{j} k}}{{\mathop \sum \nolimits_{j = 1}^{r} N_{ij} }} $$

32 $$ B_{A} = \frac{{\exp \left[ { - \mathop \sum \nolimits_{j = 1}^{r} d_{j} k\left( {p_{ij}^{*} \ln p_{ij}^{*} } \right)} \right]}}{k} $$

Here, $k$ represents the number of categories, and $d_{j}$ values were assigned based on habitat area. Alatalo’s approach highlights that different resource characteristics can be considered in weighting resource states.

For the calculation of $\beta_{i}$, $\beta_{i}^{\prime}$, $\gamma_{ih}$ and $\gamma_{ih}^{\prime}$, other options include assigning equal weighting to all factors $\left( {unw} \right)$ as suggested by Colwell and Futuyma (1971) or manually assigning weighting factors to each resource state according to user-defined criteria (Alatalo 1981).

## New Weighting Factors

In this study, we developed new weighting factors $\left( {ed_{j} ,{ }e\delta_{j} {\text{ and }}e\delta_{j}^{*} } \right)$ by modifying the original factors $\left( {d_{j} ,{ }\delta_{j} {\text{ and }}\delta_{j}^{*} } \right)$ proposed by Colwell and Futuyma (1971) and Hanski (1978) for niche metric calculations. These factors were tested using hypothetical resource matrices. The new factors are defined as:

33 $$ ed_{j} = {{\exp \left( {\delta_{j} \times \frac{{r^{\prime}}}{r}} \right)} \mathord{\left/ {\vphantom {{\exp \left( {\delta_{j} \times \frac{{r^{\prime}}}{r}} \right)} {\mathop \sum \limits_{j = 1}^{r} \exp \left( {\delta_{j} \times \frac{{r^{\prime}}}{r}} \right)}}} \right. \kern-0pt} {\mathop \sum \limits_{j = 1}^{r} \exp \left( {\delta_{j} \times \frac{{r^{\prime}}}{r}} \right)}} $$

34 $$ e\delta_{j} = ed_{j} \times \mathop \sum \limits_{j = 1}^{r} \delta_{j} $$

35 $$ e\delta_{j}^{*} = {{\exp \left( {\delta_{j}^{*} \times \frac{{r^{\prime}}}{r}} \right)} \mathord{\left/ {\vphantom {{\exp \left( {\delta_{j}^{*} \times \frac{{r^{\prime}}}{r}} \right)} {\mathop \sum \limits_{i = 1}^{r} \exp \left( {\delta_{j}^{*} \times \frac{{r^{\prime}}}{r}} \right)}}} \right. \kern-0pt} {\mathop \sum \limits_{i = 1}^{r} \exp \left( {\delta_{j}^{*} \times \frac{{r^{\prime}}}{r}} \right)}}\;{\mathrm{where}}\;\delta_{j}^{*} = {{\left( {\delta_{j} /X_{j} } \right)} \mathord{\left/ {\vphantom {{\left( {\delta_{j} /X_{j} } \right)} {\mathop \sum \limits_{i = 1}^{r} \left( {\delta_{j} /X_{j} } \right)}}} \right. \kern-0pt} {\mathop \sum \limits_{i = 1}^{r} \left( {\delta_{j} /X_{j} } \right)}} $$

Here, $r^{\prime}$ is the number of resource states occupied by the species, and $r$ is the total number of resource states. By construction, the sums of the factors satisfy $\mathop \sum \nolimits_{j = 1}^{r} d_{j} { } = \mathop \sum \nolimits_{j = 1}^{r} ed_{j} = \mathop \sum \nolimits_{j = 1}^{r} unw{ } = 1{ }$ and $\mathop \sum \nolimits_{j = 1}^{r} \delta_{j} { } = \mathop \sum \nolimits_{j = 1}^{r} { } = \mathop \sum \nolimits_{j = 1}^{r} \delta_{j}^{*} = \mathop \sum \nolimits_{j = 1}^{r} e\delta_{j} { } = \mathop \sum \nolimits_{j = 1}^{r} e\delta_{j}^{*} { }$. Notably, if ∀ $p_{ij} = N_{ij} /Z$, then $e\delta_{j} = ed_{j} = e\delta_{j}^{*} = 1/r$. Further details on the application of these new factors are provided in subsequent sections.

## Niche Metric Equations Based on Weighting Factors Other than ${\boldsymbol{d}}_{{\boldsymbol{j}}}$

If we denote all weighting factors other than $d_{j}$ $\left( {\delta_{j} ,{ }\delta_{j}^{*} ,{ }ed_{j} , e\delta_{j} ,{ }e\delta_{j}^{*} } \right)$ by the symbol $F_{j}$, the niche metrics defined by Colwell and Futuyma (1971) and Clarke (1977) can be expressed as:

36 $$ \beta_{i} = \left[ {\frac{1}{{\mathop \sum \nolimits_{j = 1}^{r} F_{j} kp_{ij}^{*2} }} - 1} \right]\left[ {\frac{1}{k - 1}} \right] $$

37 $$ \beta_{i}^{\prime} = - \frac{k}{\ln k}\mathop \sum \limits_{j = 1}^{r} F_{j} \left( {p_{ij}^{*} \ln p_{ij}^{*} } \right) $$

38 $$ \gamma_{ih} = \mathop \sum \limits_{j = 1}^{r} F_{j} - \frac{1}{2}\mathop \sum \limits_{j = 1}^{r} F_{j} k\left| {p_{ij}^{*} - p_{hj}^{*} } \right| $$

39 $$ \gamma_{ih}^{\prime} = - \frac{1}{2\ln 2}\mathop \sum \limits_{j = 1}^{r} F_{j} k\left[ {I\left( {p_{ij}^{*} } \right) + I\left( {p_{hj}^{*} } \right) - I\left( {t_{j}^{*} } \right)} \right] $$

40 $$ W_{i}^{\prime} = \exp \left( { - \mathop \sum \limits_{j = 1}^{r} F_{j} p_{ij}^{*} \ln p_{ij}^{*} } \right) $$

41 $$ O_{ih}^{\prime} = \mathop \sum \limits_{j = 1}^{r} F_{j} - \frac{1}{2}\mathop \sum \limits_{j = 1}^{r} F_{j} \left| {p_{ij}^{*} - p_{hj}^{*} } \right| $$

Equations (36)–(41) define niche metrics calculated based on the weighting factor $F_{j}$. Within the niche overlap metrics (Eqs. 38–41), the calculations of $p_{ij}^{*}$ and $p_{hj}^{*}$ are performed with respect to $d_{j}$ when $d_{j}$, $\delta_{j}$ or $\delta_{j}^{*}$ is selected as the weighting factor, and with respect to $ed_{j}$ when $ed_{j}$, $e\delta_{j}$ or $e\delta_{j}^{*}$ is chosen.

## Noncircularity in Niche Metric Calculations

Niche breadth and overlap calculations using data from all species in the resource matrix can lead to circularity. To avoid this, the niche breadth of a species should be calculated using factor values derived from a resource matrix excluding that species, and niche overlap between a species pair should use factor values calculated from a matrix excluding that species pair (Colwell and Futuyma 1971).

## Data

In the present study, eight hypothetical resource matrices (Table 2) were constructed and employed to evaluate the performance of the newly proposed weighting factors ($ed_{j}$, $e\delta_{j}$, and $e\delta_{j}^{*}$). In addition, an ecological resource matrix (Table 3) was utilized to calculate the niche metrics based on the $ed_{j}$ factor.

**Table 2:** Hypothetical resource matrices

Matrix A							Matrix B						
	R1	R2	R3					R1	R2	R3			
Sp. 1	50	30	10				Sp. 1	200	120	40			
Sp. 2	10	30	50				Sp. 2	10	30	50			
Sp. 3	5	30	55				Sp. 3	25	15	5			

Matrix C							Matrix D						
	R1	R2	R3	R4				R1	R2	R3	R4		
Sp. 1	30	30	30	0			Sp. 1	75	45	15	0		
Sp. 2	10	30	50	0			Sp. 2	10	30	50	0		
Sp. 3	5	0	55	0			Sp. 3	25	15	5	0		
Sp. 4	30	30	30	0			Sp. 4	75	45	15	0		

Matrix E							Matrix F						
	R1	R2	R3	R4				R1	R2	R3	R4		
Sp. 1	10	10	10	10			Sp. 1	40	0	0	0		
Sp. 2	0	10	10	20			Sp. 2	0	10	10	20		
Sp. 3	0	0	10	30			Sp. 3	0	0	10	30		
Sp. 4	0	0	0	40			Sp. 4	0	0	0	40		

Matrix G							Matrix H						
	R1	R2	R3	R4	R5	R6		R1	R2	R3	R4	R5	R6
Sp. 1(G)	10	10	10	10	10	10	Sp. 1(H)	10	10	10	0	0	0
Sp. 2(G)	10	0	10	10	0	10	Sp. 2(H)	10	0	10	0	0	0
Sp. 3(G)	0	0	10	0	0	10	Sp. 3(H)	0	0	10	0	0	0
Sp. 4(G)	10	0	10	10	0	10	Sp. 4(H)	10	0	10	0	0	0

**Table 3:** Ecological resource matrix: abundance of Raphignathoidea mites (Acari) in moss–lichen mixed samples collected from 13 sites across six habitats (Ceylan et al. 2022)

	SOIL	STONE	BETULA	CRATAEGUS	SALIX	QUERCUS
*Calhad*	0	0	0	0	0	5
*Neoter*	2	24	1	0	0	17
*Crylag*	0	6	0	0	0	5
*Favros*	0	0	0	0	4	0
*Rapgra*	0	2	0	0	0	19
*Rapkuz*	0	1	0	0	0	0
*Cheocc*	0	0	0	0	6	0
*Eusana*	0	1	0	0	0	5
*Eusdog*	1	0	0	0	0	0
*Euspin*	1	0	0	0	0	0
*Eusseg*	1	0	0	0	0	2
*Eustur*	0	0	0	0	0	1
*Ledayy*	2	0	0	0	0	3
*Ledtol*	2	0	0	0	0	0
*Medaeg*	0	0	0	0	0	1
*Stidev*	0	0	0	0	0	2
*Stigla*	0	0	2	0	0	0
*Storob*	0	0	0	0	0	5

See Supplementary Table 1 for the full species names

The ecological resource matrix comprises 18 raphignathoid mite species, identified and quantified from moss–lichen mixed samples collected across six habitats (bare soil (SOIL), rocky surface (STONE), birch forest (BETULA), hawthorn bush (CRATAEGUS), willow forest (SALIX), and oak forest (QUERCUS)). The scientific names of the species are provided in Supplementary Table 1, while their codes and the distribution of abundance values across habitats are presented in Table 3 (Ceylan et al. 2022).

## Results

In the hypothetical resource matrices A, B, C, and D, only the species-specific values of $p_{ij}$ and the circular weighting factors were calculated. In matrices E, F, G, and H, niche breadth was estimated using the $\beta_{i}^{\prime}$ formula (Eq. 22, $k = 10000$), while in matrix H, niche overlap was assessed based on the $\gamma_{ih}^{\prime}$ formula (Eq. 26). For the ecological resource matrix, niche breadth was computed using the $W_{i}^{\prime}$ formula (Eq. 28), and niche overlap was determined using the $O_{ih}^{\prime}$ formula (Eq. 29). Furthermore, correlation coefficients were calculated between the weighting factors and the total values of resource states (i.e., resource use or abundance) for both the hypothetical and ecological resource matrices. The results derived from the hypothetical resource matrices are presented under three subsections, whereas the results pertaining to the ecological resource matrix and the correlation analyses are provided under separate headings.

## Results of Matrices A, B, C, and D

The results for the hypothetical resource matrices A, B, C, and D are presented in Table 4. In all matrices, $\mathop \sum \nolimits_{j = 1}^{r} d_{j} { } = \mathop \sum \nolimits_{j = 1}^{r} ed_{j} = \mathop \sum \nolimits_{j = 1}^{r} unw{ } = 1$. For matrices A through D, the values of $\mathop \sum \nolimits_{j = 1}^{r} \delta_{j} { } = \mathop \sum \nolimits_{j = 1}^{r} \delta_{j}^{*} = \mathop \sum \nolimits_{j = 1}^{r} e\delta_{j} = \mathop \sum \nolimits_{j = 1}^{r} e\delta_{j}^{*} { }$ were calculated as 0.161, 0.096, 0.152, and 0.135, respectively. In matrices A and B, the values of $d_{j} \left( {R2} \right) = \delta_{j} \left( {R2} \right) = \delta_{j}^{*} \left( {R2} \right) = 0$. This occurs because in this resource state, all species have equal $p_{ij}$ values. However, as seen in Table 4, this condition does not hold for the newly proposed indices. In matrices A and B, the $e\delta_{j}$, $ed_{j}$ and $e\delta_{j}^{*}$ values for R2 are positive numbers.

**Table 4:** The $p_{ij}$ values of species in the hypothetical resource matrices A, B, C, and D, together with the results of the circular weighting factors

	Matrix A				Matrix B				
	R1	R2	R3			R1	R2	R3	
Sp. 1	0.556	0.333	0.111		Sp. 1	0.556	0.333	0.111	
Sp. 2	0.111	0.333	0.556		Sp. 2	0.111	0.333	0.556	
Sp. 3	0.056	0.333	0.611		Sp. 3	0.556	0.333	0.111	
$d_{j}$	0.576	0.000	0.424		$d_{j}$	0.422	0.000	0.578	
$\delta_{j}$	0.092	0.000	0.068		$\delta_{j}$	0.041	0.000	0.056	
$unw$	0.333	0.333	0.333		$unw$	0.333	0.333	0.333	
$\delta_{j}^{*}$	0.113	0.000	0.047		$\delta_{j}^{*}$	0.022	0.000	0.074	
$e\delta_{j}$	0.056	0.051	0.054		$e\delta_{j}$	0.032	0.031	0.033	
$ed_{j}$	0.346	0.316	0.338		$ed_{j}$	0.336	0.323	0.341	
$e\delta_{j}^{*}$	0.057	0.051	0.053		$e\delta_{j}^{*}$	0.032	0.031	0.033	

	Matrix C				Matrix D				
	R1	R2	R3	R4		R1	R2	R3	R4
Sp. 1	0.333	0.333	0.333	0.000	Sp. 1	0.556	0.333	0.111	0.000
Sp. 2	0.111	0.333	0.556	0.000	Sp. 2	0.111	0.333	0.556	0.000
Sp. 3	0.083	0.000	0.917	0.000	Sp. 3	0.556	0.333	0.111	0.000
Sp. 4	0.333	0.333	0.333	0.000	Sp. 4	0.556	0.333	0.111	0.000
$d_{j}$	0.250	0.419	0.331	0.000	$d_{j}$	0.432	0.000	0.568	0.000
$\delta_{j}$	0.032	0.053	0.042	0.000	$\delta_{j}$	0.047	0.000	0.062	0.000
$unw$	0.250	0.250	0.250	0.250	$unw$	0.250	0.250	0.250	0.250
$\delta_{j}^{*}$	0.042	0.059	0.025	0.000	$\delta_{j}^{*}$	0.028	0.000	0.081	0.000
$e\delta_{j}$	0.031	0.032	0.032	0.031	$e\delta_{j}$	0.028	0.027	0.028	0.027
$ed_{j}$	0.250	0.254	0.252	0.244	$ed_{j}$	0.254	0.245	0.257	0.245
$e\delta_{j}^{*}$	0.032	0.032	0.031	0.031	$e\delta_{j}^{*}$	0.027	0.027	0.028	0.027

In matrix C, resource state R2 contains three species, all with equal $p_{ij}$ values. Nevertheless, $d_{j} \left( {R2} \right)$, $\delta_{j} \left( {R2} \right)$ and $\delta_{j}^{*} \left( {R2} \right)$ assume distinct positive values. This is because species 3 also occurs in other resource states of matrix C. This implies that, for a given resource state, the condition $d_{j} \left( {R2} \right) = \delta_{j} \left( {R2} \right) = \delta_{j}^{*} \left( {R2} \right) = 0$ holds only if all species present in the resource matrix have identical $p_{ij}$ values in that state. Unlike matrices A and B, matrix C also includes an additional resource state, R4. R4 represents a sampled resource state in which all species have zero resource values. As shown in the factor results of matrix C (Table 4), $d_{j} \left( {R4} \right) = \delta_{j} \left( {R4} \right) = \delta_{j}^{*} \left( {R4} \right) = 0$. In other words, $d_{j}$, $\delta_{j}$ and $\delta_{j}^{*}$ exclude resource states with a total resource value of zero. By contrast, the newly proposed factors behave differently. For R4 in matrix C, the computed values of $e\delta_{j}$, $ed_{j}$ and $e\delta_{j}^{*}$ are distinct positive numbers.

Matrix D, similar to matrix C, includes four resource states. In R4, all species have zero resource values, whereas in R2, all species occur and share identical $p_{ij}$ values. Consequently, both R2 and R4 have $d_{j} = \delta_{j} = \delta_{j}^{*} = 0$. The results for R2 indicate that if all species in the resource matrix occur in the $j$th resource state and their $p_{ij}$ values are equal, then $d_{j} = \delta_{j} = \delta_{j}^{*} = 0$. The same applies to R4, where the absence of any resource value for all species $\left( {\forall p_{ij} = 0} \right)$ also yields neutral values.

In summary, the neutral values of $d_{j}$, $\delta_{j}$ and $\delta_{j}^{*}$ correspond to the lowest values for a given resource state. Therefore, the newly proposed factors are also expected to yield the lowest outputs under these conditions. The results in Table 4 confirm this: R2 in matrices A and B, R4 in matrix C, and R2 and R4 in matrix D exhibit the lowest values of $e\delta_{j}$, $ed_{j}$ and $e\delta_{j}^{*}$ compared to the other resource states within the same matrices.

## Results of Matrices E and F

In matrices E and F, niche breadth calculations were conducted based on both circular and noncircular weighting factors: $d_{j}$, $\delta_{j}$, $\delta_{j}^{*}$, $ed_{j}$, $e\delta_{j}$, and $e\delta_{j}^{*}$. The circular and noncircular values of these weighting factors for matrices E and F are provided in Supplementary Tables 2, 3, 4, and 5.

In matrix E, the resource states with the highest circular weighting factor values are R1 and R2 (Supplementary Table 2). In this matrix, the highest noncircular values of the weighting factors vary depending on the species excluded: when Sp. 1 is excluded, the maximum occurs in R2; when Sp. 2 is excluded, in R1 and R2; and when species 3 or 4 are excluded, in R1 (Supplementary Table 3). In matrix F, the resource state with the highest circular weighting factors is R1 (Supplementary Table 4). For the noncircular case, the maximum values are observed in R2 when Sp. 1 is excluded, and in R1 when Sp. 2, Sp. 3, or Sp. 4 are excluded (Supplementary Table 5).

Depending on the selected factor, the circular niche breadth values of species may be equal to, larger than, or smaller than their noncircular counterparts. For example, in matrix E, the circular and noncircular niche breadth values of Sp. 1 are identical when calculated with $d_{j}$ and $ed_{j}$, whereas for other factors, the circular values are greater than the noncircular values. For Sp. 3, the circular niche breadth values are greater than the noncircular ones when calculated using $d_{j}$, $\delta_{j}$, $\delta_{j}^{*}$, and $ed_{j}$; however, for $e\delta_{j}$ and $e\delta_{j}^{*}$, the circular values are smaller (Table 5).

**Table 5:** Circular and noncircular $\beta_{i}^{\prime}$ values of species in resource matrices E and F

Matrix E								
	Circular $\beta_{i}^{\prime}$				Noncircular $\beta_{i}^{\prime}$			
Factors	Sp. 1	Sp. 2	Sp. 3	Sp. 4	Sp. 1	Sp. 2	Sp. 3	Sp. 4
$d_{j}$	1.000	0.956	0.890	0.839	1.000	0.959	0.909	0.833
$\delta_{j}$	0.863	0.818	0.753	0.701	0.854	0.841	0.769	0.640
$\delta_{j}^{*}$	0.863	0.770	0.647	0.521	0.854	0.824	0.702	0.492
$e\delta_{j}$	0.863	0.825	0.772	0.711	0.854	0.845	0.771	0.656
$ed_{j}$	1.000	0.962	0.909	0.849	1.000	0.962	0.910	0.849
$e\delta_{j}^{*}$	0.863	0.822	0.768	0.706	0.854	0.843	0.767	0.653

Matrix F								
	Circular $\beta_{i}^{\prime}$				Noncircular $\beta_{i}^{\prime}$			
Factors	Sp. 1	Sp. 2	Sp. 3	Sp. 4	Sp. 1	Sp. 2	Sp. 3	Sp. 4
$d_{j}$	0.923	0.920	0.886	0.854	ud	0.916	0.895	0.851
$\delta_{j}$	0.874	0.871	0.837	0.805	ud	0.890	0.855	0.789
$\delta_{j}^{*}$	0.842	0.897	0.792	0.684	ud	0.909	0.849	0.726
$e\delta_{j}$	0.817	0.907	0.858	0.801	0.696	0.928	0.868	0.788
$ed_{j}$	0.866	0.956	0.907	0.850	0.842	0.954	0.908	0.849
$e\delta_{j}^{*}$	0.808	0.911	0.853	0.789	0.696	0.932	0.867	0.781

*ud* undefined

Noncircular niche breadth results could be obtained for all species in matrix E. However, a special case prevents the estimation of noncircular values when $d_{j}$, $\delta_{j}$ or $\delta_{j}^{*}$ are used as weighting factors. This case is exemplified in matrix F. In this matrix, species 1 and species 4 are represented by data from only a single resource state, and their values in those states are identical. The only difference is that the resource state containing Sp. 1 includes no other species, whereas the resource state containing Sp. 4 includes all species.

For all species in matrix F, circular niche breadth values can be calculated across all factors. However, when using noncircular $d_{j}$, $\delta_{j}$, or $\delta_{j}^{*}$ as weighting factors, the niche breadth of Sp. 1 becomes undefined. This occurs because in R1, the noncircular values of $d_{j}$, $\delta_{j}$, or $\delta_{j}^{*}$ are zero, resulting in $Y_{sp1}^{*} = 0$ and $p_{ij}^{*} = N_{{{\mathrm{Sp}}.{ }1{\mathrm{R}}1}} /0$, which is mathematically undefined. This limitation represents the major drawback of using $d_{j}$, $\delta_{j}$, or $\delta_{j}^{*}$ as weighting factors.

By contrast, this special case does not affect niche breadth calculations based on the noncircular factors $ed_{j}$, $e\delta_{j}$, or e $\delta_{j}^{*}$. When these factors are used, niche breadth values are meaningful for all species. Interestingly, the results obtained with circular $ed_{j}$, $e\delta_{j} ,$ or e $\delta_{j}^{*}$ indicate that the niche breadth of Sp. 1 is larger than that of Sp. 4. This is unexpected, since Sp. 1 occurs in only one resource state that contains no other species, whereas Sp. 4 occurs in a state with three additional species. Clearly, R1 represents the most specialized resource in matrix F. Therefore, Sp. 1 should have a narrower niche breadth than species 4. Indeed, when the noncircular $ed_{j}$, $e\delta_{j}$, or e $\delta_{j}^{*}$ factors are applied, this expectation is met: Sp. 1 receives the narrowest niche breadth value, as it should (Table 5).

## Results of Matrices G and H

Hypothetical matrices G and H were designed to enable a comparison of species niche breadths. For this comparison, it was assumed that both matrices contain the same resource states and the same species. In matrix G, all six resource states include at least one species. In each resource state, the abundance values of all species are equal, and R1, R2, and R3 contain the same species as R4, R5, and R6, respectively. Similarly, in matrix H, the species composition and their resource use values in R1, R2, and R3 are identical to those in R1, R2, and R3 of matrix G. However, the remaining resource states in matrix H do not include any species-specific resource use data.

The circular and noncircular values of the weighting factors calculated for matrices G and H are provided in Supplementary Tables 6, 7, 8, and 9. In matrix G, the highest circular weighting factor values are observed in resource states R2 and R5 (Supplementary Table 6). The highest noncircular values occur in R1 and R5 when Sp. 1 is excluded, and in R2 when Sp. 2, Sp. 3 or Sp. 4 are excluded (Supplementary Table 7). In matrix H, the highest circular weighting factors are found in R2 (Supplementary Table 8). The highest noncircular values occur in R1 when Sp. 1 is excluded, and in R2 when any of the other species are excluded (Supplementary Table 9).

As shown in Table 6, species in both matrices have identical niche breadth values when calculated with the $d_{j}$ factor. This outcome occurs because, as previously noted, $d_{j} = 0$ for resource states without resource values. Clearly, niche breadth values based on $d_{j}$ are unsuitable for comparing matrices that share the same resource states. Similarly, results obtained with $\delta_{j}$ and $\delta_{j}^{*}$ are also unsatisfactory, since all species have lower niche breadth values in matrix G than in matrix H. The results of $e\delta_{j}$ and $e\delta_{j}^{*}$ do not fully meet expectations either, as the niche breadth of Sp. 1 in matrix G is lower than that of species 1 in matrix H.

**Table 6:** Noncircular $\beta_{i}^{\prime}$ values of species in hypothetical resource matrices G and H

	Matrix G				Matrix H			
Factors	Sp. 1	Sp. 2	Sp. 3	Sp. 4	Sp. 1	Sp. 2	Sp. 3	Sp. 4
$d_{j}$	1.000	0.925	0.693	0.925	1.000	0.925	0.693	0.925
$\delta_{j}$	0.735	0.708	0.420	0.708	0.811	0.764	0.477	0.764
$\delta_{j}^{*}$	0.735	0.653	0.310	0.653	0.811	0.709	0.367	0.709
$e\delta_{j}$	0.735	0.738	0.606	0.738	0.738	0.721	0.589	0.721
$ed_{j}$	1.000	0.955	0.880	0.955	0.926	0.882	0.805	0.882
$e\delta_{j}^{*}$	0.735	0.738	0.606	0.738	0.738	0.720	0.588	0.720

By contrast, the results based on $ed_{j}$ align with expectations. According to this factor, the niche breadth values of species in matrix G are greater than those of their counterparts in matrix H. Moreover, within a single resource matrix, $ed_{j}$ is the most appropriate factor for comparing noncircular niche breadth values among species. In matrix G, for instance, Sp. 1 has the largest niche breadth value, followed by Sp. 2 and Sp. 4, which share the same value. However, when $e\delta_{j}^{*}$ or $e\delta_{j}$ are used, Sp. 2 and Sp. 4 appear to have larger noncircular niche breadth values than Sp. 1 (Table 6).

Niche overlap calculations were conducted using the data from Matrix H. With the exception of the species pairs Sp. 1 & Sp. 2 and Sp. 1 & Sp. 4, the highest values of all factors were associated with resource state R1. For the species pairs Sp. 2 & Sp. 3, Sp. 2 & Sp. 4, and Sp. 3 & Sp. 4, the highest values were associated with R2. When the species pair Sp. 1 & Sp. 3 was excluded, the values of the $d_{j}$, $\delta_{j}$ and $\delta_{j}^{*}$ factors were zero across all resource states, whereas the values of the $ed_{j}$, $e\delta_{j}$ and $e\delta_{j}^{*}$ factors were 0.167 (see Supplementary Table 10).

The noncircular $\gamma_{ih}^{\prime}$ results of species pairs, based on weight factors, are presented in Table 7. For the species pair Sp. 1 & Sp. 3, the niche overlap calculations according to $d_{j}$, $\delta_{j}$ and $\delta_{j}^{*}$ were undefined. By contrast, the novel factors ($ed_{j}$, $e\delta_{j}$ and $e\delta_{j}^{*}$) yielded positive values for all species pairs. However, according to $e\delta_{j}$ and $e\delta_{j}^{*}$, the Sp. 1 & Sp. 3 pair exhibited the highest niche overlap. In contrast, according to $ed_{j}$, the highest overlap value was found in the Sp. 2 & Sp. 4 pair, as expected, since both species occurred in the same resource states with the same relative resource values.

**Table 7:** Noncircular $\gamma_{ih}^{\prime}$ values of species pairs in matrices G and H

Factors	Species pairs					
	Sp, 1&Sp, 2	Sp, 1&Sp, 3	Sp, 1&Sp, 4	Sp, 2&Sp, 3	Sp, 2&Sp, 4	Sp, 3&Sp, 4
$d_{j}$	1.000	ud	1.000	0.689	1.000	0.689
$\delta_{j}$	0.274	ud	0.274	0.077	0.208	0.077
$\delta_{j}^{*}$	0.274	ud	0.274	0.042	0.18	0.042
$e\delta_{j}$	0.224	0.541	0.224	0.077	0.208	0.077
$ed_{j}$	0.819	0.541	0.819	0.689	1.000	0.689
$e\delta_{j}^{*}$	0.225	0.541	0.225	0.077	0.207	0.077

*ud* undefined

In summary, as in the case of niche breadth, only the $ed_{j}$ factor produced results consistent with theoretical expectations in niche overlap calculations.

## Results of the Ecological Resource Matrix

In the ecological resource matrix, for the computation of noncircular niche breadth and niche overlap, the formulas $W_{i}^{\prime}$ (Eq. 28) and $\gamma_{ih}^{\prime}$ (Eq. 26) were employed instead of the formulas $\beta_{i}^{\prime}$ (Eq. 22) and $O_{ih}^{\prime}$ (Eq. 29) used in the hypothetical resource matrices.

Among the species in the ecological resource matrix, one species occurs in four habitats, five species occur in two habitats, and the remaining species are found in only one habitat. SALIX exhibits the highest $ed_{j}$ value (0.178), followed by STONE (0.172). The $ed_{j}$ values of QUERCUS, SOIL, and BETULA are 0.168, 0.167, and 0.161, respectively. CRATAEGUS, which shows the lowest $ed_{j}$ (0.154), harbors no species.

According to $W_{i}^{\prime}$ results given in Table 8, the species with the broadest niche breadth are, in order: *Neoter*, *Crylag*, *Ledayy*, *Eusseg*, *Eusana*, and *Rapgra*. Species *Calhad*, *Favros*, *Rapkuz*, *Cheocc*, *Eusdog*, *Euspin*, *Eustur*, *Ledtol*, *Medaeg*, *Stidev*, *Stigla*, and *Storob* occur in only one habitat, with individual counts ranging from 1 to 6. For species in a single habitat, $p_{ij} = 1$. Among these, *Stigla* in BETULA (with 2 individuals) has the narrowest niche breadth (0.155). Its low niche breadth arises from co-occurrence with *Neoter*, the species with the largest niche breadth in the ecological matrix. In BETULA, *Neoter* has the lowest relative abundance $\left( {p_{ij} = 0.023} \right)$, which results in *Stigla* having the largest noncircular $p_{ij}^{*} = 6.431$. Similarly, *Ledtol*, present only in SOIL (2 individuals), has the second-largest noncircular $p_{ij}^{*}$ and the second-smallest niche breadth (0.165). *Eusdog* and *Euspin* co-occur with *Ledtol* in SOIL but are single individuals. Their noncircular $p_{ij}^{*}$ values are 6.019, yielding niche breadth values (0.166) slightly larger than *Ledtol*’s.

**Table 8:** Noncircular $p_{ij}^{*}$ values for species present in a single habitat and noncircular $W_{i}^{\prime}$ values for all species

Species	Noncircular $p_{ij}^{*}$	Noncircular $W_{i}^{\prime}$
*Calhad*	5.954	0.168
*Neoter*		0.423
*Crylag*		0.339
*Favros*	5.772	0.173
*Rapgra*		0.231
*Rapkuz*	5.840	0.171
*Cheocc*	5.888	0.170
*Eusana*		0.266
*Eusdog*	6.019	0.166
*Euspin*	6.019	0.166
*Eusseg*		0.317
*Eustur*	5.946	0.168
*Ledayy*		0.328
*Ledtol*	6.070	0.165
*Medaeg*	5.946	0.168
*Stidev*	5.948	0.168
*Stigla*	6.431	0.155
*Storob*	5.954	0.168

Table 8 presents the $p_{ij}^{*}$ values of the species occurring in a single habitat along with their corresponding $W_{i}^{\prime}$ values. Spearman correlation analysis between them revealed an exceptionally strong negative relationship $\left( {{\mathrm{r}} = - 0.999} \right)$, demonstrating that as the $p_{ij}^{*}$ value of a species increases, its $W_{i}^{\prime}$ value decreases almost perfectly. The entirety of the finding indicates that: (1) a species’ niche breadth in a single habitat depends on the $p_{ij}$ values of co-occurring species, (2) a species with more individuals than another species in the same habitat is more dependent on that habitat, and 3) the greater a species’ dependence on a habitat, the smaller its niche breadth.

The noncircular niche overlap values for all possible species pairs are presented in Supplementary Table 11. Fourteen species pairs have a value of 1, all of which consist of species restricted to a single habitat and sharing that habitat. These are followed by the pairs *Eusseg & Ledayy*, *Neoter & Crylag*, and *Rapgra & Eusana*, with niche overlap values of 0.934, 0.929, and 0.927, respectively. In contrast, species pairs occurring in completely different habitats have a niche overlap value of 0, with 80 pairs falling into this category.

## Relationships Between Resource State Abundance and Weighting Factors

Table 9 presents the results of correlation analyses between weighting factors and the total abundance values of resource states $\left( {X_{j} } \right)$ for hypothetical resource matrices and the ecological resource matrix. Negative relationships between $X_{j}$ and weighting factors were observed in Matrices A, B, E, F, and G. In Matrix G, all factors exhibited very strong correlations with $X_{j}$, whereas in Matrices A, B, E, and F, the relationships of $X_{j}$ with the $\delta_{j}^{*}$ and $e\delta_{j}^{*}$ factors were stronger than those with the other factors.

**Table 9:** Correlation analyses between weighting factors and the total abundance of resource states $\left( {X_{j} } \right)$ in hypothetical resource matrices (A, B, C, D, E, F, G, and H) and the ecological resource matrix $\left( {{\mathrm{ER}}} \right)$

Matrices/factors	$d_{j}$	$\delta_{j}$	$\delta_{j}^{*}$	$ed_{j}$	$e\delta_{j}$	$e\delta_{j}^{*}$
A	−0.254	−0.254	−0.581	−0.264	−0.264	−0.594
B	−0.261	−0.261	−0.686	−0.267	−0.267	−0.692
C	0.649	0.649	0.288	0.643	0.643	0.283
D	0.433	0.433	0.255	0.431	0.431	0.250
E	−0.310	−0.310	−0.587	−0.318	−0.318	−0.585
F	0.330	0.330	−0.470	0.305	0.305	−0.471
G	−0.999	−0.999	−0.986	−0.999	−0.999	−0.986
H	0.485	0.485	0.153	0.478	0.478	0.145
ER	0.421	0.421	−0.472	0.416	0.416	−0.472

In Matrix H, the correlation coefficients were positive. Compared to $\delta_{j}^{*}$ and $e\delta_{j}^{*}$, the relationships of $X_{j}$ with the $d_{j}$, $\delta_{j}$, $ed_{j}$ and $e\delta_{j}$ factors were stronger.

According to the results for the ecological resource matrix, $X_{j}$ is positively correlated with the weighting factors $d_{j}$, $\delta_{j}$, $ed_{j}$, and $e\delta_{j}$, but negatively correlated with, $\delta_{j}^{*}$ and $e\delta_{j}^{*}$, with the latter showing a stronger relationship with $X_{j}$ than the other factors.

## Discussion and Conclusion

The paper by Colwell and Futuyma (1971), “On the Measurement of Niche Breadth and Overlap,” presents formulas and example solutions for niche metrics ($\beta_{i}$, $\beta_{i}^{\prime}$, $\gamma_{ih}$, and $\gamma_{ih}^{\prime}$) based on information theory. This paper has been cited more than 2,300 times according to Google Scholar data (accessed 2025) and is considered a classic in the ecological literature (Loreau 1990). However, to our knowledge, very few of these citations involve the direct use of the original niche metrics as proposed by the authors (Rolando 1990; Inger and Colwell 1977; Heyer 1974; Sabath and Jones 1973). This is largely due to two major criticisms concerning the weighting factors used in the niche calculations, as raised by Hanski (1978) and Loreau (1990).

Hanski (1978) argued that the $d_{j}$ or $\delta_{j}$ values assigned to resource states are highly correlated with their abundances and proposed a new weighting factor $\left( {\delta_{j}^{*} } \right)$. However, this evaluation was not based on a simulation study but on results obtained using hypothetical resource matrices generated by Hanski (1978), Colwell and Futuyma’s (1971) hypothetical matrices, and the fertilizer beetle resource matrices from Hanski and Koskela (1977). The correlation analyses between the weighting factors and the total abundances $\left( {X_{j} } \right)$ for each of the hypothetical resource matrices A, B, C, D, E, F, G, H given in Table 2 and ecological resource matrix, ER given in Table 3 presented in this study, do not correspond with the results obtained in Hanski’s (1978) own study.

As shown in Table 9, the correlation coefficients between $d_{j}$ or $\delta_{j}$ and $X_{j}$ can be lower than those between $\delta_{j}^{*}$ and $X_{j}$. In other words, the relationship between $d_{j}$ or $\delta_{j}$ and $X_{j}$ is not “typical.” Moreover, the correlations between the modified factors and $X_{j}$ are also not typical, as the correlation coefficients of $d_{j}$, $\delta_{j}$, $ed_{j}$ and $e\delta_{j}$ are very similar to each other, whereas the correlation coefficients of $e\delta_{j}^{*}$ closely match those of $\delta_{j}^{*}$.

Loreau (1990) critically evaluated Colwell and Futuyma’s (1971) metrics, focusing particularly on $d_{j}$ and $\delta_{j}$, using two hypothetical resource matrices. His critique included Hanski’s proposed factor $\left( {\delta_{j}^{*} } \right)$. In both of the resource matrices provided by Loreau (1990), there are two species and three resource states. In the first resource matrix, all resource states have the same total value, and the values of the species in the second resource state are identical. In the second resource matrix, however, the total values of the three resource states and the values of the species in the second resource state differ from one another. Using the equations provided by Colwell and Futuyma (1971), Loreau (1990) demonstrated that the $M_{j} \left( X \right) = \delta_{j}$ values for the second resource states in both matrices are neutral. The researcher noted that it is reasonable for the $\delta_{j}$ value of the second resource state in the first matrix to be neutral, because all three species have the same resource values in that state; however, it is illogical for the $\delta_{j}$ value of the second resource state in the second matrix to be neutral, since the species’ resource values differ in that state. According to Loreau (1990), the neutral $\delta_{j}$ value in the second resource state of the second matrix represents a fundamental problem for niche metric calculations, and this issue propagates into the matrix expansion step, further increasing deviations in niche metric estimates.

The niche metric calculations proposed by Colwell and Futuyma (1971) are based on the relative abundances of species within resource states. In contrast, the critique by Loreau (1990) focuses on the actual values of species in the resource states. In both hypothetical resource matrices provided by Loreau (1990), the proportional values of the species in the second resource state are equal. Therefore, according to the formulas given by Colwell and Futuyma (1971), it is not unexpected that the $\delta_{j}$ values are neutral (zero).

Loreau’s (1990) critique regarding unequal values in resource states could be considered valid if the species had identical morphological, anatomical, functional, and genetic traits. However, morphological, anatomical, functional, and genetic characteristics are already the universal criteria used to distinguish species. Therefore, the critique by Loreau (1990) concerning the $\delta_{j}$ value of the second resource state in the second matrix essentially amounts to the same issue as the critique of the $\delta_{j}$ value of the second resource state in the first matrix.

In our view, the $\delta_{j}$ values of the second resource states in both resource matrices provided by Loreau (1990) can indeed be neutral, and a neutral $\delta_{j}$ implies that $d_{j}$ would also be neutral. However, the $F_{j}$ values given in Eqs. (36)–(41) must be positive for all resource states. Otherwise, calculating niche metrics under all conditions would be impossible. An example of this can be seen in the niche breadth calculations for species in Matrix F. In Matrix F, the noncircular $d_{j}$, $\delta_{j}$, and $\delta_{j}^{*}$ values for species Sp. 1 are undefined. In contrast, the noncircular $ed_{j}$, $e\delta_{j}$, and $e\delta_{j}^{*}$ values for Sp. 1 in the same matrix correspond to positive numerical results. In summary, regardless of the characteristics of the resource matrices, using the modified factors allows noncircular niche metric calculations for all species. Furthermore, the use of modified weighting factors $\left( {ed_{j} ,{ }e\delta_{j} ,{\text{ and }}e\delta_{j}^{*} } \right)$ also prevents an increase in the bias of niche metric calculations caused by matrix expansion. Colwell and Futuyma (1971) suggested that the expanded resource matrix constant could be $k = 10,000$, although depending on the structure of the resource matrix, $k$ may take a lower value. Sabath and Jones (1973) calculated species’ niche metrics using different $k$ values. Their results showed that while niche metric values change with different $k$ values, the relative ranking of species’ niche breadths remains unaffected. Matrix expansion mainly ensures that $\beta_{i}$ or $\beta_{i}^{\prime}$ values are calculated as positive. Even if the ranking of niche breadth values remains unchanged, using very large $k$ values reduces the visible differences between the niche breadth values. Clarke (1977) calculated species’ niche breadths using the $d_{j}$ factor from the formulas proposed by Colwell and Futuyma (1971) but without applying the $k$ constant. The niche breadth formula proposed by Clarke (1977) eliminates the need for matrix expansion. Accordingly, Clarke’s (1977) niche breadth formula also renders Loreau’s (1990) criticism regarding error increase from matrix expansion in Colwell and Futuyma’s (1971) niche breadth calculations largely irrelevant.

Some resource states in the resource matrix may contain no numerical data. If the number of such resource states is high, significant deviations can occur in niche metric estimates based on the weighting factors $d_{j}$, $\delta_{j}$, and $\delta_{j}^{*}$. This is because for resource states with no values, $d_{j} = \delta_{j} = \delta_{j}^{*} = 0$ It should be noted that even if a resource state contains numerical values, $d_{j} = \delta_{j} = \delta_{j}^{*} = 0$ can still occur. Such a result is possible if a resource state includes all species in the resource matrix and the relative values of these species in that resource state are equal. However, when the modified factors $ed_{j}$, $e\delta_{j}$, and $e\delta_{j}^{*}$ are used in niche metric calculations, this problem does not occur. With these factors, resource states containing no numerical values and those containing all species with equal relative values are assigned the smallest positive values (see Matrices C and D).

In Matrix H, three resource states contain no numerical values. Therefore, for these resource states, $d_{j} = \delta_{j} = \delta_{j}^{*} = 0$, which is reflected in the niche breadth calculations for the species. Indeed, based on the $d_{j}$, $\delta_{j}$, and $\delta_{j}^{*}$ factors, it is not possible to assign ecological meaning to the niche breadth results of species in Matrix H. The absence of numerical values for three resource states in Matrix H, and the resulting $d_{j} = \delta_{j} = \delta_{j}^{*} = 0$, also prevents a meaningful comparison of species’ niche breadths between Matrices G and H. Additionally, for some species pairs in Matrix H, noncircular niche overlap calculations based on $d_{j}$, $\delta_{j}$, and $\delta_{j}^{*}$ are undefined. In contrast, noncircular niche overlap calculations based on the modified factors $ed_{j}$, $e\delta_{j}$, and $e\delta_{j}^{*}$ yield numerical results for all species pairs. Using $ed_{j}$, $e\delta_{j}$, and $e\delta_{j}^{*}$ ensures that noncircular niche breadth and overlap values can be calculated for any species or species pair in all types of resource matrices. However, this does not mean that all three modified factors necessarily produce ecologically meaningful results. Indeed, the niche metric results for Matrices G and H show that, compared to $e\delta_{j}$ and $e\delta_{j}^{*}$, the most ecologically sensible results are obtained using the $ed_{j}$ factor.

The validation of the $ed_{j}$ factor in niche metric calculations was conducted using a real resource matrix composed of mite species (ecological resource matrix). According to the simple niche breadth formulas (Eqs. 1, 2), all species occurring in a single habitat receive the minimum niche breadth value. This value corresponds to 1 with respect to Levins’ index $\left( {B_{i} } \right)$, and to 0 with respect to Shannon entropy $\left( {B_{i}^{\prime} } \right)$. However, calculations based on the $ed_{j}$ factor assign different noncircular niche breadth values to species in a single habitat, and, as expected, these values are lower than the noncircular niche breadth values of species occurring in multiple habitats. Noncircular niche overlap values between species pairs also yield ecologically reasonable results.

The results obtained from the resource matrices used in this study indicate that ecologically meaningful niche breadth and niche overlap values can be obtained when the $ed_{j}$ factor is applied in the formulas of Colwell and Futuyma (1971) $\left( {\beta_{i} ,{ }\beta_{i}^{\prime} ,{ }\gamma_{ih} ,{ }\gamma_{ih}^{\prime} { }} \right)$ or Clarke (1977) ($W_{i}^{\prime}$ and $O_{ih}^{\prime}$). However, the $ed_{j}$ factor should be further tested in niche metric calculations across different species groups and resource matrices of varying sizes and characteristics, and, if necessary, strengthened with new modifications.

## Supplementary Information

Below is the link to the electronic supplementary material.

Supplementary file1 (DOCX 87 KB)

## Data Availability

Data will be made available on reasonable request.
