# Supplementary material for: Refining Niche Metric Calculations: A Modified Weighting Approach to Colwell and Futuyma’s Method
Source: Bull Math Biol. 2026 Jun 12;88(7):115. doi: 10.1007/s11538-026-01672-w (PMC13263204; doi:10.1007/s11538-026-01672-w)
Supplement: Supplementary file 1 — Supplementary file1 (DOCX 87 KB) [file 11538_2026_1672_MOESM1_ESM.docx]

**Supplementary Tables**

Supplementary Table 1. List of raphignathoid mite species and their corresponding codes documented in the ecological data matrix, sampled from six distinct macro-habitat types in the Pülümür Valley (Türkiye) and adjacent areas (Ceylan et al., 2022)

| Species | Codes |
| --- | --- |
| *Caligonella haddadi* Bagheri & Maleki | *Calhad* |
| *Neognathus terrestris* (Summers & Schlinger) | *Neoter* |
| *Cryptognathus lagena* Kramer | *Crylag* |
| *Favognathus rosulatus* Doğan & Doğan | *Favros* |
| *Raphignathus gracilis* (Rack) | *Rapgra* |
| *Raphignathus kuznetzovi* Doğan & Ayyıldız | *Rapkuz* |
| *Cheylostigmaeus occultatus* Doğan & Doğan | *Cheocc* |
| *Eustigmaeus anauniensis* (Canestrini) | *Eusana* |
| *Eustigmaeus dogani* Khanjani, Fayaz, Mirmoayedi & Ghaedi | *Eusdog* |
| *Eustigmaeus pinnatus* (Kuznetsov) | *Euspin* |
| *Eustigmaeus segnis* (Koch) | *Eusseg* |
| *Eustigmaeus turcicus* Doğan & Ayyıldız | *Eustur* |
| *Ledermuelleriopsis ayyildizi* Doğan | *Ledayy* |
| *Ledermuelleriopsis toleratus* Kuznetsov | *Ledtol* |
| *Mediolata aegyptiaca* (Zaher & Soliman) | *Medaeg* |
| *Stigmaeus devlethanensis* Akyol & Koç | *Stidev* |
| *Stigmaeus glabrisetus* Summers | *Stigla* |
| *Storchia robusta* (Berlese) | *Storob* |

Supplementary Table 2. Circular values of weighting factors assigned to resource states in Matrix E (see Table 2 in the main text for Matrix E)

| Weighting factors | Resource states | | | |
| --- | --- | --- | --- | --- |
|  | R1 | R2 | R3 | R4 |
| $d_{j}$ | 0.295 | 0.295 | 0.184 | 0.226 |
| $\delta_{j}$ | 0.083 | 0.083 | 0.052 | 0.064 |
| $\delta_{j}^{*}$ | 0.158 | 0.079 | 0.033 | 0.012 |
| ${e\delta}_{j}$ | 0.071 | 0.071 | 0.069 | 0.070 |
| ${ed}_{j}$ | 0.253 | 0.253 | 0.245 | 0.248 |
| ${e\delta}_{j}^{*}$ | 0.077 | 0.071 | 0.068 | 0.066 |

Supplementary Table 3. Noncircular values of weighting factors for resource states in Matrix E, calculated by excluding each species in turn (see Table 2 in the main text for Matrix E)

| Excluded species | Weighting factors | Resource states | | | |  |  |
| --- | --- | --- | --- | --- | --- | --- | --- |
|  |  | R1 | R2 | R3 | R4 |  |  |
|  | $d_{j}$ | 0.000 | 0.488 | 0.361 | 0.151 |  |  |
|  | $\delta_{j}$ | 0.000 | 0.127 | 0.094 | 0.039 |  |  |
|  | $\delta_{j}^{*}$ | 0.000 | 0.185 | 0.068 | 0.006 |  |  |
| Sp. 1 | ${e\delta}_{j}$ | 0.061 | 0.073 | 0.065 | 0.061 |  |  |
|  | ${ed}_{j}$ | 0.234 | 0.266 | 0.257 | 0.243 |  |  |
|  | ${e\delta}_{j}^{*}$ | 0.061 | 0.073 | 0.065 | 0.061 |  |  |
|  |  |  |  |  |  |  |  |
|  |  | R1 | R2 | R3 | R4 |  |  |
|  |  | 0.274 | 0.274 | 0.203 | 0.248 |  |  |
|  | $d_{j}$ | 0.093 | 0.093 | 0.069 | 0.084 |  |  |
|  | $\delta_{j}$ | 0.137 | 0.137 | 0.050 | 0.015 |  |  |
|  | $\delta_{j}^{*}$ | 0.086 | 0.086 | 0.083 | 0.085 |  |  |
| Sp. 2 | ${e\delta}_{j}$ | 0.252 | 0.252 | 0.246 | 0.250 |  |  |
|  | ${ed}_{j}$ | 0.089 | 0.089 | 0.082 | 0.079 |  |  |
|  | ${e\delta}_{j}^{*}$ | 0.252 | 0.252 | 0.246 | 0.250 |  |  |
|  |  |  |  |  |  |  |  |
|  |  | R1 | R2 | R3 | R4 |  |  |
|  | $d_{j}$ | 0.295 | 0.218 | 0.218 | 0.269 |  |  |
|  | $\delta_{j}$ | 0.082 | 0.060 | 0.060 | 0.075 |  |  |
|  | $\delta_{j}^{*}$ | 0.148 | 0.055 | 0.055 | 0.019 |  |  |
| Sp. 3 | ${e\delta}_{j}$ | 0.070 | 0.069 | 0.069 | 0.070 |  |  |
|  | ${ed}_{j}$ | 0.253 | 0.248 | 0.248 | 0.251 |  |  |
|  | ${e\delta}_{j}^{*}$ | 0.075 | 0.068 | 0.068 | 0.066 |  |  |
|  |  |  |  |  |  |  |  |
|  |  | R1 | R2 | R3 | R4 |  |  |
|  | $d_{j}$ | 0.452 | 0.333 | 0.000 | 0.215 |  |  |
|  | $\delta_{j}$ | 0.076 | 0.056 | 0.000 | 0.036 |  |  |
|  | $\delta_{j}^{*}$ | 0.117 | 0.043 | 0.000 | 0.009 |  |  |
| Sp. 4 | ${e\delta}_{j}$ | 0.044 | 0.043 | 0.041 | 0.042 |  |  |
|  | ${ed}_{j}$ | 0.259 | 0.254 | 0.240 | 0.248 |  |  |
|  | ${e\delta}_{j}^{*}$ | 0.046 | 0.042 | 0.041 | 0.041 |  |  |

Supplementary Table 4. Circular values of weighting factors for resource states in Matrix F (see Table 2 in the main text for Matrix F)

| Weighting | Resource states | | | |
| --- | --- | --- | --- | --- |
| factors | R1 | R2 | R3 | R4 |
| $d_{j}$ | 0.493 | 0.123 | 0.123 | 0.260 |
| $\delta_{j}$ | 0.314 | 0.079 | 0.079 | 0.166 |
| $\delta_{j}^{*}$ | 0.233 | 0.233 | 0.116 | 0.055 |
| ${e\delta}_{j}$ | 0.185 | 0.146 | 0.146 | 0.160 |
| ${ed}_{j}$ | 0.290 | 0.230 | 0.230 | 0.250 |
| ${e\delta}_{j}^{*}$ | 0.171 | 0.171 | 0.152 | 0.143 |

Supplementary Table 5. Noncircular values of weighting factors for resource states in Matrix F, calculated by excluding each species in turn (see Table 2 in the main text for Matrix F)

| Excluded species | Weighting factors | Resource states | | | |  |  |
| --- | --- | --- | --- | --- | --- | --- | --- |
|  |  | R1 | R2 | R3 | R4 |  |  |
|  | $d_{j}$ | 0.000 | 0.488 | 0.361 | 0.151 |  |  |
|  | $\delta_{j}$ | 0.000 | 0.127 | 0.094 | 0.039 |  |  |
|  | $\delta_{j}^{*}$ | 0.000 | 0.185 | 0.068 | 0.006 |  |  |
| Sp. 1 | ${e\delta}_{j}$ | 0.061 | 0.069 | 0.067 | 0.063 |  |  |
|  | ${ed}_{j}$ | 0.234 | 0.266 | 0.257 | 0.243 |  |  |
|  | ${e\delta}_{j}^{*}$ | 0.061 | 0.073 | 0.065 | 0.061 |  |  |
|  |  |  |  |  |  |  |  |
|  |  | R1 | R2 | R3 | R4 |  |  |
|  | $d_{j}$ | 0.523 | 0.000 | 0.131 | 0.346 |  |  |
|  | $\delta_{j}$ | 0.413 | 0.000 | 0.103 | 0.273 |  |  |
|  | $\delta_{j}^{*}$ | 0.332 | 0.000 | 0.332 | 0.126 |  |  |
| Sp. 2 | ${e\delta}_{j}$ | 0.242 | 0.160 | 0.177 | 0.210 |  |  |
|  | ${ed}_{j}$ | 0.306 | 0.203 | 0.225 | 0.266 |  |  |
|  | ${e\delta}_{j}^{*}$ | 0.223 | 0.160 | 0.223 | 0.182 |  |  |
|  |  |  |  |  |  |  |  |
|  |  | R1 | R2 | R3 | R4 |  |  |
|  | $d_{j}$ | 0.469 | 0.117 | 0.117 | 0.296 |  |  |
|  | $\delta_{j}$ | 0.325 | 0.081 | 0.081 | 0.205 |  |  |
|  | $\delta_{j}^{*}$ | 0.202 | 0.202 | 0.202 | 0.085 |  |  |
| Sp. 3 | ${e\delta}_{j}$ | 0.200 | 0.157 | 0.157 | 0.178 |  |  |
|  | ${ed}_{j}$ | 0.289 | 0.227 | 0.227 | 0.257 |  |  |
|  | ${e\delta}_{j}^{*}$ | 0.178 | 0.178 | 0.178 | 0.158 |  |  |
|  |  |  |  |  |  |  |  |
|  |  | R1 | R2 | R3 | R4 |  |  |
|  | $d_{j}$ | 0.521 | 0.130 | 0.096 | 0.252 |  |  |
|  | $\delta_{j}$ | 0.296 | 0.074 | 0.055 | 0.143 |  |  |
|  | $\delta_{j}^{*}$ | 0.206 | 0.206 | 0.076 | 0.080 |  |  |
| Sp. 4 | ${e\delta}_{j}$ | 0.165 | 0.132 | 0.130 | 0.142 |  |  |
|  | ${ed}_{j}$ | 0.290 | 0.232 | 0.228 | 0.249 |  |  |
|  | ${e\delta}_{j}^{*}$ | 0.151 | 0.151 | 0.133 | 0.133 |  |  |

Supplementary Table 6. Circular values of weighting factors for resource states in Matrix G (see Table 2 in the main text for Matrix G)

| Weighting | Resource states | | | | | |
| --- | --- | --- | --- | --- | --- | --- |
| factors | R1 | R2 | R3 | R4 | R5 | R6 |
| $d_{j}$ | 0.133 | 0.284 | 0.083 | 0.133 | 0.284 | 0.083 |
| $\delta_{j}$ | 0.017 | 0.037 | 0.011 | 0.017 | 0.037 | 0.011 |
| $\delta_{j}^{*}$ | 0.008 | 0.053 | 0.004 | 0.008 | 0.053 | 0.004 |
| ${e\delta}_{j}$ | 0.166 | 0.169 | 0.165 | 0.166 | 0.169 | 0.165 |
| ${ed}_{j}$ | 0.021 | 0.022 | 0.021 | 0.021 | 0.022 | 0.021 |
| ${e\delta}_{j}^{*}$ | 0.021 | 0.022 | 0.021 | 0.021 | 0.022 | 0.021 |

Supplementary Table 7. Noncircular values of weighting factors for resource states in Matrix G, calculated by excluding each species in turn (see Table 2 in the main text for Matrix G)

| Excluded species | Weighting factors | Resource states | | | | | |
| --- | --- | --- | --- | --- | --- | --- | --- |
|  |  | R1 | R2 | R3 | R4 | R5 | R6 |
|  | $d_{j}$ | 0.377 | 0.000 | 0.123 | 0.377 | 0.000 | 0.123 |
|  | $\delta_{j}$ | 0.033 | 0.000 | 0.011 | 0.033 | 0.000 | 0.011 |
|  | $\delta_{j}^{*}$ | 0.036 | 0.000 | 0.008 | 0.036 | 0.000 | 0.008 |
| Sp. 1 | ${e\delta}_{j}$ | 0.015 | 0.014 | 0.014 | 0.015 | 0.014 | 0.014 |
|  | ${ed}_{j}$ | 0.170 | 0.164 | 0.166 | 0.170 | 0.164 | 0.166 |
|  | ${e\delta}_{j}^{*}$ | 0.015 | 0.014 | 0.014 | 0.015 | 0.014 | 0.014 |
|  |  |  |  |  |  |  |  |
|  |  | R1 | R2 | R3 | R4 | R5 | R6 |
|  | $d_{j}$ | 0.146 | 0.250 | 0.104 | 0.146 | 0.250 | 0.104 |
|  | $\delta_{j}$ | 0.020 | 0.034 | 0.014 | 0.020 | 0.034 | 0.014 |
|  | $\delta_{j}^{*}$ | 0.014 | 0.047 | 0.007 | 0.014 | 0.047 | 0.007 |
| Sp. 2 | ${e\delta}_{j}$ | 0.023 | 0.023 | 0.022 | 0.023 | 0.023 | 0.022 |
|  | ${ed}_{j}$ | 0.166 | 0.169 | 0.165 | 0.166 | 0.169 | 0.165 |
|  | ${e\delta}_{j}^{*}$ | 0.022 | 0.023 | 0.022 | 0.022 | 0.023 | 0.022 |
|  |  |  |  |  |  |  |  |
|  |  | R1 | R2 | R3 | R4 | R5 | R6 |
|  | $d_{j}$ | 0.030 | 0.441 | 0.030 | 0.030 | 0.441 | 0.030 |
|  | $\delta_{j}$ | 0.002 | 0.036 | 0.002 | 0.002 | 0.036 | 0.002 |
|  | $\delta_{j}^{*}$ | 0.001 | 0.039 | 0.001 | 0.001 | 0.039 | 0.001 |
| Sp. 3 | ${e\delta}_{j}$ | 0.013 | 0.014 | 0.013 | 0.013 | 0.014 | 0.013 |
|  | ${ed}_{j}$ | 0.165 | 0.170 | 0.165 | 0.165 | 0.170 | 0.165 |
|  | ${e\delta}_{j}^{*}$ | 0.013 | 0.014 | 0.013 | 0.013 | 0.014 | 0.013 |
|  |  |  |  |  |  |  |  |
|  |  | R1 | R2 | R3 | R4 | R5 | R6 |
|  | $d_{j}$ | 0.146 | 0.250 | 0.104 | 0.146 | 0.250 | 0.104 |
|  | $\delta_{j}$ | 0.020 | 0.034 | 0.014 | 0.020 | 0.034 | 0.014 |
|  | $\delta_{j}^{*}$ | 0.014 | 0.047 | 0.007 | 0.014 | 0.047 | 0.007 |
| Sp. 4 | ${e\delta}_{j}$ | 0.023 | 0.023 | 0.022 | 0.023 | 0.023 | 0.022 |
|  | ${ed}_{j}$ | 0.166 | 0.169 | 0.165 | 0.166 | 0.169 | 0.165 |
|  | ${e\delta}_{j}^{*}$ | 0.022 | 0.023 | 0.022 | 0.022 | 0.023 | 0.022 |

Supplementary Table 8. Circular values of weighting factors for resource states in Matrix H (see Table 2 in the main text for Matrix H)

| Weighting | Resource states | | | | | |
| --- | --- | --- | --- | --- | --- | --- |
| factors | R1 | R2 | R3 | R4 | R5 | R6 |
| $d_{j}$ | 0.265 | 0.568 | 0.167 | 0.000 | 0.000 | 0.000 |
| $\delta_{j}$ | 0.059 | 0.126 | 0.037 | 0.000 | 0.000 | 0.000 |
| $\delta_{j}^{*}$ | 0.028 | 0.180 | 0.013 | 0.000 | 0.000 | 0.000 |
| ${e\delta}_{j}$ | 0.168 | 0.174 | 0.167 | 0.164 | 0.164 | 0.164 |
| ${ed}_{j}$ | 0.037 | 0.039 | 0.037 | 0.036 | 0.036 | 0.036 |
| ${e\delta}_{j}^{*}$ | 0.037 | 0.040 | 0.036 | 0.036 | 0.036 | 0.036 |

Supplementary Table 9. Noncircular values of weighting factors for resource states in Matrix H, calculated by excluding each species in turn (see Table 2 in the main text for Matrix H)

| Excluded species | Weighting factors | Resource states | | | | | |
| --- | --- | --- | --- | --- | --- | --- | --- |
|  |  | R1 | R2 | R3 | R4 | R5 | R6 |
|  | $d_{j}$ | 0.753 | 0.000 | 0.247 | 0.000 | 0.000 | 0.000 |
|  | $\delta_{j}$ | 0.133 | 0.000 | 0.043 | 0.000 | 0.000 | 0.000 |
|  | $\delta_{j}^{*}$ | 0.145 | 0.000 | 0.032 | 0.000 | 0.000 | 0.000 |
| Sp. 1 | ${e\delta}_{j}$ | 0.031 | 0.029 | 0.030 | 0.029 | 0.029 | 0.029 |
|  | ${ed}_{j}$ | 0.175 | 0.164 | 0.168 | 0.164 | 0.164 | 0.164 |
|  | ${e\delta}_{j}^{*}$ | 0.031 | 0.029 | 0.029 | 0.029 | 0.029 | 0.029 |
|  |  |  |  |  |  |  |  |
|  |  | R1 | R2 | R3 | R4 | R5 | R6 |
|  | $d_{j}$ | 0.292 | 0.500 | 0.208 | 0.000 | 0.000 | 0.000 |
|  | $\delta_{j}$ | 0.067 | 0.114 | 0.047 | 0.000 | 0.000 | 0.000 |
|  | $\delta_{j}^{*}$ | 0.047 | 0.160 | 0.022 | 0.000 | 0.000 | 0.000 |
| Sp. 2 | ${e\delta}_{j}$ | 0.039 | 0.040 | 0.038 | 0.037 | 0.037 | 0.037 |
|  | ${ed}_{j}$ | 0.169 | 0.173 | 0.167 | 0.163 | 0.163 | 0.163 |
|  | ${e\delta}_{j}^{*}$ | 0.038 | 0.040 | 0.038 | 0.037 | 0.037 | 0.037 |
|  |  |  |  |  |  |  |  |
|  |  | R1 | R2 | R3 | R4 | R5 | R6 |
|  | $d_{j}$ | 0.059 | 0.881 | 0.059 | 0.000 | 0.000 | 0.000 |
|  | $\delta_{j}$ | 0.008 | 0.121 | 0.008 | 0.000 | 0.000 | 0.000 |
|  | $\delta_{j}^{*}$ | 0.003 | 0.131 | 0.003 | 0.000 | 0.000 | 0.000 |
| Sp. 3 | ${e\delta}_{j}$ | 0.023 | 0.024 | 0.023 | 0.023 | 0.023 | 0.023 |
|  | ${ed}_{j}$ | 0.165 | 0.175 | 0.165 | 0.165 | 0.165 | 0.165 |
|  | ${e\delta}_{j}^{*}$ | 0.023 | 0.024 | 0.023 | 0.023 | 0.023 | 0.023 |
|  |  |  |  |  |  |  |  |
|  |  | R1 | R2 | R3 | R4 | R5 | R6 |
|  | $d_{j}$ | 0.292 | 0.500 | 0.208 | 0.000 | 0.000 | 0.000 |
|  | $\delta_{j}$ | 0.067 | 0.114 | 0.047 | 0.000 | 0.000 | 0.000 |
|  | $\delta_{j}^{*}$ | 0.047 | 0.160 | 0.022 | 0.000 | 0.000 | 0.000 |
| Sp. 4 | ${e\delta}_{j}$ | 0.039 | 0.040 | 0.038 | 0.037 | 0.037 | 0.037 |
|  | ${ed}_{j}$ | 0.039 | 0.040 | 0.038 | 0.037 | 0.037 | 0.037 |
|  | ${e\delta}_{j}^{*}$ | 0.169 | 0.173 | 0.167 | 0.163 | 0.163 | 0.163 |

Supplementary Table 10. Noncircular values of weighting factors for resource states in Matrix H, calculated by excluding each species pair in turn (see Table 2 in the main text for Matrix H)

| Sp. 1 & Sp. 2 | R1 | R2 | R3 | R4 | R5 | R6 |
| --- | --- | --- | --- | --- | --- | --- |
| $d_{j}$ | 0.775 | 0.000 | 0.225 | 0.000 | 0.000 | 0.000 |
| $\delta_{j}$ | 0.212 | 0.000 | 0.062 | 0.000 | 0.000 | 0.000 |
| $\delta_{j}^{*}$ | 0.239 | 0.000 | 0.035 | 0.000 | 0.000 | 0.000 |
| ${e\delta}_{j}$ | 0.050 | 0.045 | 0.046 | 0.045 | 0.045 | 0.045 |
| ${ed}_{j}$ | 0.181 | 0.163 | 0.168 | 0.163 | 0.163 | 0.163 |
| ${e\delta}_{j}^{*}$ | 0.050 | 0.045 | 0.045 | 0.045 | 0.045 | 0.045 |
|  |  |  |  |  |  |  |
| Sp. 1 & Sp. 3 | R1 | R2 | R3 | R4 | R5 | R6 |
| $d_{j}$ | 0.000 | 0.000 | 0.000 | 0.000 | 0.000 | 0.000 |
| $\delta_{j}$ | 0.000 | 0.000 | 0.000 | 0.000 | 0.000 | 0.000 |
| $\delta_{j}^{*}$ | 0.000 | 0.000 | 0.000 | 0.000 | 0.000 | 0.000 |
| ${e\delta}_{j}$ | 0.167 | 0.167 | 0.167 | 0.167 | 0.167 | 0.167 |
| ${ed}_{j}$ | 0.167 | 0.167 | 0.167 | 0.167 | 0.167 | 0.167 |
| ${e\delta}_{j}^{*}$ | 0.167 | 0.167 | 0.167 | 0.167 | 0.167 | 0.167 |
|  |  |  |  |  |  |  |
| Sp. 1 & Sp. 4 | R1 | R2 | R3 | R4 | R5 | R6 |
| $d_{j}$ | 0.775 | 0.000 | 0.225 | 0.000 | 0.000 | 0.000 |
| $\delta_{j}$ | 0.212 | 0.000 | 0.062 | 0.000 | 0.000 | 0.000 |
| $\delta_{j}^{*}$ | 0.239 | 0.000 | 0.035 | 0.000 | 0.000 | 0.000 |
| ${e\delta}_{j}$ | 0.050 | 0.045 | 0.046 | 0.045 | 0.045 | 0.045 |
| ${ed}_{j}$ | 0.181 | 0.163 | 0.168 | 0.163 | 0.163 | 0.163 |
| ${e\delta}_{j}^{*}$ | 0.050 | 0.045 | 0.045 | 0.045 | 0.045 | 0.045 |
|  |  |  |  |  |  |  |
| Sp. 2 & Sp. 3 | R1 | R2 | R3 | R4 | R5 | R6 |
| $d_{j}$ | 0.069 | 0.862 | 0.069 | 0.000 | 0.000 | 0.000 |
| $\delta_{j}$ | 0.008 | 0.097 | 0.008 | 0.000 | 0.000 | 0.000 |
| $\delta_{j}^{*}$ | 0.004 | 0.104 | 0.004 | 0.000 | 0.000 | 0.000 |
| ${e\delta}_{j}$ | 0.019 | 0.019 | 0.019 | 0.019 | 0.019 | 0.019 |
| ${ed}_{j}$ | 0.166 | 0.173 | 0.166 | 0.165 | 0.165 | 0.165 |
| ${e\delta}_{j}^{*}$ | 0.019 | 0.020 | 0.019 | 0.019 | 0.019 | 0.019 |
|  |  |  |  |  |  |  |
| Sp. 2 & Sp. 4 | R1 | R2 | R3 | R4 | R5 | R6 |
| $d_{j}$ | 0.333 | 0.333 | 0.333 | 0.000 | 0.000 | 0.000 |
| $\delta_{j}$ | 0.069 | 0.069 | 0.069 | 0.000 | 0.000 | 0.000 |
| $\delta_{j}^{*}$ | 0.083 | 0.083 | 0.042 | 0.000 | 0.000 | 0.000 |
| ${e\delta}_{j}$ | 0.035 | 0.035 | 0.035 | 0.034 | 0.034 | 0.034 |
| ${ed}_{j}$ | 0.170 | 0.170 | 0.170 | 0.164 | 0.164 | 0.164 |
| ${e\delta}_{j}^{*}$ | 0.035 | 0.035 | 0.035 | 0.034 | 0.034 | 0.034 |
|  |  |  |  |  |  |  |
| Sp. 3 & Sp. 4 | R1 | R2 | R3 | R4 | R5 | R6 |
| $d_{j}$ | 0.069 | 0.862 | 0.069 | 0.000 | 0.000 | 0.000 |
| $\delta_{j}$ | 0.008 | 0.097 | 0.008 | 0.000 | 0.000 | 0.000 |
| $\delta_{j}^{*}$ | 0.004 | 0.104 | 0.004 | 0.000 | 0.000 | 0.000 |
| ${e\delta}_{j}$ | 0.019 | 0.019 | 0.019 | 0.019 | 0.019 | 0.019 |
| ${ed}_{j}$ | 0.166 | 0.173 | 0.166 | 0.165 | 0.165 | 0.165 |
| ${e\delta}_{j}^{*}$ | 0.019 | 0.020 | 0.019 | 0.019 | 0.019 | 0.019 |

Supplementary Table 11. Noncircular niche overlap values calculated using the $O_{ih}^{'}$ formula

|  | *Calhad* | *Neoter* | *Crylag* | *Favros* | *Rapgra* | *Rapkuz* | *Cheocc* | *Eusana* | *Eusdog* | *Euspin* | *Eusseg* | *Eustur* | *Ledayy* | *Ledtol* | *Medaeg* | *Stidev* | *Stigla* | *Storob* |
| --- | --- | --- | --- | --- | --- | --- | --- | --- | --- | --- | --- | --- | --- | --- | --- | --- | --- | --- |
| *Calhad* |  |  |  |  |  |  |  |  |  |  |  |  |  |  |  |  |  |  |
| *Neoter* | 0.394 |  |  |  |  |  |  |  |  |  |  |  |  |  |  |  |  |  |
| *Crylag* | 0.452 | 0.929 |  |  |  |  |  |  |  |  |  |  |  |  |  |  |  |  |
| *Favros* | 0.000 | 0.000 | 0.000 |  |  |  |  |  |  |  |  |  |  |  |  |  |  |  |
| *Rapgra* | 0.903 | 0.485 | 0.546 | 0.000 |  |  |  |  |  |  |  |  |  |  |  |  |  |  |
| *Rapkuz* | 0.000 | 0.537 | 0.549 | 0.000 | 0.098 |  |  |  |  |  |  |  |  |  |  |  |  |  |
| *Cheocc* | 0.000 | 0.000 | 0.000 | 1.000 | 0.000 | 0.000 |  |  |  |  |  |  |  |  |  |  |  |  |
| *Eusana* | 0.831 | 0.556 | 0.619 | 0.000 | 0.927 | 0.170 | 0.000 |  |  |  |  |  |  |  |  |  |  |  |
| *Eusdog* | 0.000 | 0.046 | 0.000 | 0.000 | 0.000 | 0.000 | 0.000 | 0.000 |  |  |  |  |  |  |  |  |  |  |
| *Euspin* | 0.000 | 0.046 | 0.000 | 0.000 | 0.000 | 0.000 | 0.000 | 0.000 | 1.000 |  |  |  |  |  |  |  |  |  |
| *Eusseg* | 0.668 | 0.441 | 0.452 | 0.000 | 0.667 | 0.000 | 0.000 | 0.669 | 0.329 | 0.329 |  |  |  |  |  |  |  |  |
| *Eustur* | 1.000 | 0.394 | 0.451 | 0.000 | 0.902 | 0.000 | 0.000 | 0.830 | 0.000 | 0.000 | 0.669 |  |  |  |  |  |  |  |
| *Ledayy* | 0.603 | 0.442 | 0.453 | 0.000 | 0.602 | 0.000 | 0.000 | 0.604 | 0.394 | 0.394 | 0.934 | 0.604 |  |  |  |  |  |  |
| *Ledtol* | 0.000 | 0.045 | 0.000 | 0.000 | 0.000 | 0.000 | 0.000 | 0.000 | 1.000 | 1.000 | 0.327 | 0.000 | 0.391 |  |  |  |  |  |
| *Medaeg* | 1.000 | 0.394 | 0.451 | 0.000 | 0.902 | 0.000 | 0.000 | 0.830 | 0.000 | 0.000 | 0.669 | 1.000 | 0.604 | 0.000 |  |  |  |  |
| *Stidev* | 1.000 | 0.394 | 0.451 | 0.000 | 0.902 | 0.000 | 0.000 | 0.830 | 0.000 | 0.000 | 0.669 | 1.000 | 0.604 | 0.000 | 1.000 |  |  |  |
| *Stigla* | 0.000 | 0.020 | 0.000 | 0.000 | 0.000 | 0.000 | 0.000 | 0.000 | 0.000 | 0.000 | 0.000 | 0.000 | 0.000 | 0.000 | 0.000 | 0.000 |  |  |
| *Storob* | 1.000 | 0.394 | 0.452 | 0.000 | 0.903 | 0.000 | 0.000 | 0.831 | 0.000 | 0.000 | 0.668 | 1.000 | 0.603 | 0.000 | 1.000 | 1.000 | 0.000 |  |
